# The concurrent validity of the Internet Addiction Test (IAT) and the Mobile Phone Dependence Questionnaire (MPDQ)

**DOI:** 10.1371/journal.pone.0197562

**Published:** 2018-06-26

**Authors:** Fung Chin, Chi Hung Leung

**Affiliations:** 1 Department of Special Education and Counselling, The Education University of Hong Kong, Hong Kong SAR, China; 2 Department of Christian Counseling, Bethel Bible Seminary, Hong Kong SAR, China; Cairnmillar Institute, AUSTRALIA

## Abstract

Internet addiction and mobile phone addiction are both forms of technology addiction, and thus would be expected to show similarities and differences. This study investigated the association between the Internet Addiction Test (IAT) and the Mobile Phone Dependence Questionnaire (MPDQ) as a test of concurrent validity. Participants were 1,072 students aged between 9 and 18 years old (63% male and 37% female) from three primary schools and three secondary schools in Hong Kong. Correlations showed that scores on the two measures were moderately correlated, providing further evidence of each measure’s validity. Confirmatory factor analysis that the IAT’s factor structure was similar in both younger and older samples, suggesting that it is developmentally appropriate for primary and secondary school students. Latent Class Analysis showed that 4 classes or 5 classes are appropriate for IAT's score classification. ROC analyses showed similar rates of participants with high scores on the IAT and on the MPDQ. The outcomes have implications for the prevention and treatment of Internet and mobile phone addiction. Future research can establish norms for different ages, genders and cultural groups.

## Introduction

Use of the Internet and smartphones has grown all over the world, with more than three billion people using the Internet daily [1] and two and half billion people using a smartphone every day [2]. Hong Kong has seen similar growth in the use of these technologies, which have become a necessity of daily life among most of Hong Kong’s residents [3,4]. In Hong Kong, the number of home computers connected to the Internet has increased by 143%, from 0.77 million (2000) to 1.8 million (2013) [5]. Meanwhile, the Office of the Communications Authority [6] reported that in 2016, there were 2.28 mobile phones per resident in Hong Kong, indicating one of the highest penetration rates of mobile phone use in the world [7]. Many of these users of the Internet and mobile phones are adolescents, who tend to rely on these technologies for entertainment more than adults do [4]. In Hong Kong it is estimated that 23% of youth aged 10 to 19 are Internet users [5] and that 18% of youth aged 10 to 19 are smart phone users [6], with the high rate of social media use in this group (91%) occurring primarily via mobile phones [8]. However, overuse of the Internet and of mobile phones is associated with problems for adolescents, whose physical health, family life, and academic performance can be particularly negatively affected [9,10,11,12]. Paradoxically, technology that is commonly used as a means of communication can also foster reduced family interaction and social life, and increased feelings of depression and loneliness.

The term addiction (or dependence) has been used to describe extreme overuse of the Internet and of mobile phones [13,14], or more specifically, overuse of certain programs or applications that these technologies afford, such as social media and texting [15]. Young people who have grown up with digital innovations are the primary users of these technologies, and they are the most likely to be described in research studies as showing Internet addiction and mobile phone addiction [7]. Ezoe, Lida, Lnoue, and Toda [16] indicated that as commonly conceptualized, mobile phone addiction is similar to Internet addiction in many aspects. In addition, smart phone technology allows the mobile phone user to access the Internet. The combination of the two technologies suggests that behaviors related to the Internet and mobile phones cannot be considered separately [17, 18]. Assessment measures are key to further research in this area. In the current study we tested whether the most commonly used measure of Internet addiction and a conceptually similar measure of mobile phone addiction were significantly correlated with each other. This information would provide evidence of the concurrent validity of each measure.

### Definitions of Internet and mobile phone addictions

It is important to note that no one is addicted to the Internet or a cell phone *per se*. It would be more accurate to say that a person is addicted to the entertainment and communication afforded by these technologies [14,15]. Nevertheless, the terms “Internet addiction” and “mobile phone addiction” are used ubiquitously in the literature. Research definitions of Internet addiction and mobile phone addiction are consistent with general conceptualizations of addiction. Some definitions refer to the inability to regulate use, resulting in negative consequences for health, social, and financial aspects of life [19, 20]. Other definitions highlight compulsive use, craving, tolerance, withdrawal and functional impairment as key characteristics [21, 22, 23]. Tolerance is likely to lead to spending increasing amounts of time on the device to achieve satisfaction [24]. Withdrawal symptoms include irritability, depression, and annoyance when the user is unable to use the Internet or mobile phone, while at the same time engaging in obsessive thinking or fantasies about using the technology to reduce withdrawal symptoms [7].

Many research definitions of Internet addiction and mobile phone addiction are based on the criteria for other disorders in the *Diagnostic and Statistical Manual of Mental Disorders*, either the 4^th^ edition (DSM-IV; American Psychiatric Association, 1994) or 5^th^ edition (DSM-5; American Psychiatric Association, 2013). Specifically, researchers have used the diagnostic criteria for substance dependence (DSM-IV) [25] or substance use disorder (DSM-5) [26] and gambling disorder (DSM-5) [22, 26] to define Internet addiction and mobile phone addiction. The DSM 5-also lists internet gaming disorder as a condition for further study, with the possibility that it would be considered a formal disorder in future editions of the DSM [26, 27].

### Prevalence of Internet addiction and mobile phone addiction in Hong Kong

Estimates of prevalence are affected by the definitions of Internet addiction and mobile phone addiction used in any given study. In addition, any given measure may not show the same psychometric properties or construct validity in different samples, such as samples from different cultures, or from different age groups. This means that the same cutoff score might not have the same meaning in the two groups. Although it would be ideal to conduct ROC curve analysis to define cutoff scores for specific populations, prevalence studies rarely do so.

In general, research suggests that both Internet addiction and mobile phone addiction are common conditions in Hong Kong. In the case of Internet addiction, the rate among adolescents may be higher than in older parts of the world. Whereas the rate of Internet addiction in secondary students has been estimated to be 4% in the US [28], 16% in Korea [29], and 8% in Taiwan and China [30], the rate has been estimated to be at least 20% in Hong Kong. In 2013 the Hong Kong Federation of Education Workers [31] stated that at least 20% of secondary school students were addicted to the Internet according to their teachers’ observations. Shek and Yu [32] reported a rate of 26.4% in a sample of 3,328 students from 28 secondary schools in Hong Kong. Rates of mobile phone addiction among adolescents in Hong Kong also appear to be high. Recently, a study of 733 students from six secondary schools in Hong Kong showed that 16.90% of the students fell into the category of high-level mobile phone dependence, with the rate being 13.80% and 22.70% for boys and girls, respectively [33].

### Internet Addiction Test (IAT) and Mobile Phone Dependence Questionnaire (MPDQ)

In this section, we provide an overview of the two measures that were analyzed in the current study. Additional information is provided in the Measures section. The Internet Addiction Test [34] is the most commonly used measure of Internet addiction [24]. Information is gathered through 20 self-report items, with addiction being defined as meeting five of seven criteria over a six-month period: obsession with the Internet, increasingly spending a long time on the Internet, having time management problems, repeated attempts to reduce Internet use, environmental distress, deception regarding time spent online, and mood modification through Internet use [28]. A Hong Kong version of the IAT was developed and validated in a Hong Kong sample and the measure showed good psychometric properties [35].

Leung [33] created the MPDQ-Hong Kong version as a screening tool for mobile phone addiction. Its criteria are similar to the *Diagnostic and Statistical Manual of Mental Disorders* (4th ed., text rev.; DSM-IV-TR) [36] criteria for substance dependence. The measure uses 20 self-report items to assess three dimensions of mobile phone addiction, based on confirmatory factor analysis: compulsive text messaging, compulsive making and receiving of calls, and distorted thinking about using mobile phones. The outcomes suggested that adolescents in Hong Kong are inclined to use the mobile phone to keep an intimate affiliation with parents and friends, and that there are gender differences in mobile phone addiction. Leung [33] also validated the measure in a Hong Kong sample.

### Present study

Both the IAT and the MPDQ have been adapted for use with Hong Kong samples, and evidence suggests that these scales are culturally suitable [33,37,38]. However, Leung [33] noted that the concurrent validity of the MPDQ with established measures of Internet addiction has not yet been documented. Given that the IAT and the MPDQ measure both similar and dissimilar features of children’s and adolescents’ Internet and mobile phone use, our aim in the current study was to test the association between these two measures in order to evaluate their concurrent validity. In addition, even though the Internet and smartphones are used by all age groups, youth are the most common users, and the most vulnerable to addiction. Consequently, the scope of the current study was limited to children and teenagers between 9 and 18 years old who studied in primary and secondary schools in Hong Kong. Given this wide age range, we conducted the analyses for the full sample and for the younger and older subsamples. This study makes a unique contribution to the literature by testing the concurrent validity of both instruments and identifying the extent to which the instruments are applicable to both younger and older students.

### Hypotheses

This study hypothesize that the two overall scores will be correlated, and that we use exploratory analyses to examine the correlations among subscales The current study proposed the following six hypotheses:

1. There will be a significant positive association between the withdrawal and social problems subscales of the IAT and the compulsive text messaging subscale of the MPDQ.
2. There will be a significant positive association between the withdrawal and social problems subscale of the IAT and the compulsive making and receiving of calls subscale of the MPDQ.
3. There will be a significant positive association between the time management and performance subscale of the IAT and the compulsive text messaging subscale of the MPDQ.
4. There will be a significant positive association between the time management and performance subscale of the IAT and the compulsive making and receiving of calls subscale of the MPDQ.
5. There will be a significant positive association between the reality substitute subscale of the IAT and the distorted thinking subscale of the MPDQ.
6. The associations among the subscales of the IAT and subscales of the MPDQ in primary school and secondary school subsamples will be similar to those found in the whole sample.

## Materials and methods

Our study has been approved by the Education University of Hong Kong Human Research Ethics Committee (HREC) and the whole research process was under its monitoring.

### Participants

A clustered random sample of 1,072 students was recruited from 18 classes from three primary schools and six secondary schools in Hong Kong between May and July 2017. During the assessment period, 1,121 pupils attended lessons on the day of data collection, and a total of 1,097 (97.9%) participated in the survey. However, 25 outliers considered impracticable were removed after data screening; therefore, only data from 1,072 students were considered valid. The mean age was 12.42 years (*SD* = 2.01), and the age range was from 9 to 18 (63% male and 37% female). There were 390 students from primary grade 5 and grade 6, and 682 from secondary grade 1 to grade 6.

### Procedure

An invitation letter was sent to numerous primary and secondary students to join the study. Students whose parents had signed the consent form were told the aim and procedures of the research study and were informed that involvement was voluntary, that they could withdraw from the study at any time, and that the data were confidential and would be coded without the use of names. The students were then asked to sign an assent form if they agreed to participate. Completion of the surveys took around 15 minutes. All data were input into SPSS 21.0 for further investigation.

### Instruments

#### Background information

Students first provided background information regarding their age, gender, average duration of mobile use per day, and the activities in which they engaged online.

#### Young’s Chinese version of the Internet Addiction Test

This 20-item questionnaire [39] measures the degree to which Internet use affects a respondent’s daily routine, social life, productivity, sleeping pattern, and feelings, based on the DSM-IV criteria for pathological gambling. Respondents use a 5-point scale ranging from 1 (*rarely*) to 5 (*always*) to report the frequency with which they engaged in listed Internet behaviors during the past year. Sample questions include “How often do you find yourself anticipating when you will go online again?”, “How often do you lose sleep due to late-night logins?”, and “How often do you feel preoccupied with the Internet when offline, or fantasize about being online?” The possible score varies from 20 to 100. Faraci, Craparo, Messina, and Severino [37] suggested that a score of 20–39 indicates an average user, 40–69 indicates a problematic user, and 70–100 indicates an addicted user.

Several studies have examined the validity and reliability of the IAT. Widyanto and McMurran [38] found that the subscales showed moderate to good internal consistency, with Cronbach’s alpha coefficients ranging from 0.54 to 0.82. Chang and Law [40] further divided the IAT into a three-factor model: (a) withdrawal and social problems, (b) time management and performance, and (c) reality substitute. The Cronbach’s alpha coefficients for the three factors ranged from .63 to .92. Withdrawal refers to feelings of difficulty and moodiness when being restrained from Internet use, and social problems describe a person using the Internet to search for social comfort and social interaction to substitute for real-life social activities [40]. Time management is related to the level of compulsive Internet use and being unable to control the sum of time spent on the Internet, and performance is related to lack of self-control and neglect of academics or work [40]. Reality substitute describes an individual perceiving the Internet as another reality and abusing it to avoid real-life issues [40].

#### Mobile Phone Dependence Questionnaire (MPDQ)

The MPDQ is a questionnaire first developed by Ezoe, Lida, Lnoue, and Toda [16] with a self-rating scale including 20 items on mobile phone dependency (e.g., “I send lots of long email messages,” “When I am riding on a train or in similar situations, I tend to handle my mobile phone,” “I would rather lose my wallet or purse than my mobile phone”). Respondents use a 4-point Likert scale ranging from 0 (*rarely*) to 3 (*always*). Item scores are then summed to create a total mobile phone dependency score ranging from 0 to 60. Higher scores indicate a larger dependency on mobile phones. The reliability coefficient (Cronbach’s alpha) for the MPDQ in the current sample was 0.86[16].

According to Leung [33], there are three psychological factors in mobile phone dependency: “compulsive text messaging,” “compulsive making and receiving of calls,” and “distorted thinking about using mobile phones.” The CFA model was shown to be theoretically appropriate and psychometrically sound, with TLI = 0.93, CFI = 0.94, and RMSEA = 0.06 *p* < .001 [33]. Regarding the reliability coefficient (Cronbach’s alpha) for the MPDQ subscales, compulsive text messaging loaded the highest (*r* = 0.87), distorted thinking was second (*r* = 0.75), and compulsive making and receiving calls was third (*r* = 0.66).

Leung [33] further explained that “compulsive text messaging” and “compulsive making and receiving of calls” referred to repetitive and compulsive behavior for no practical reason:

Such behaviour is similar to what has been called ‘technological addiction’, which is thought to involve excessive human–machine interactions that develop when people rely on the device to provide psychological benefits, such as an expected reduction in negative mood states or an expected increase in positive outcomes [33].

Finally, distorted thinking about using mobile phones could be related to compulsive and persistent thinking about using cell phones. According to Brown [41], distorted thinking is defined as the cognitive factor in behavioral addiction in that the activity controls the individual’s thoughts.

## Results

### Internal consistency

Our first step was to verify that the two measures had adequate internal consistency. We tested the omega reliability for the MPDQ, the IAT, and their subscales (see Table 1). It is known that coefficient alpha underestimates the true reliability unless the items are tau-equivalent, and coefficient omega is deemed to be a practical alternative to coefficient alpha in estimating measurement reliability. Using a confirmatory factor analysis, the omega reliability for the overall MPDQ score was .91, and for the overall IAT score, 0.90.

**Table 1:** Omega reliabilities for MPDQ and IAT total scores and three subscales (N = 1072).

	G	F1	F2	F3
MPDQ	.91	.85	.83	.70
IAT	.90	.81	.82	.65

F1 = Compulsive text messaging and time management and performance; F2 = Compulsive making and receiving of calls and withdrawal and social problem; F3 = Distorted thinking and Reality substitute; MPDQ = Mobile Phone Dependence Questionnaire, IAT = Internet Addiction Test.

### Descriptive statistics

Tables 2 and 3 show the means and standard deviations of the IAT and MPDQ total scores as well as the subscales of each measure.

**Table 2:** Means and standard deviations of the IAT (N = 1072).

	IAT total score	Withdrawal and social problems	Time management and performance	Reality substitute
Mean	34.3	15.21	9.01	4.46
SD	10.7	5.42	3.18	1.85

IAT = Internet Addiction Test, SD = standard deviation.

**Table 3:** Means and standard deviations of the MPDQ (N = 1072).

	MPDQ total score	Compulsive text messaging	Compulsive making and receiving of calls	Distorted thinking
Mean	34.2	13.07	13.01	8.11
SD	9.8	4.48	4.42	2.96

MPDQ = Mobile Phone Dependence Questionnaire, SD = standard deviation.

### Correlations

The Pearson correlation coefficients showed significant associations between the IAT and MPDQ total scores and among the subscales of each measure (see Table 4). Analyses regarding specific hypotheses are discussed below.

**Table 4:** Correlation matrix of IAT and MPDQ total scores and subscale scores (N = 1072).

	1	2	3	4	5	6	7	8	9
1. IAT total score	1								
2. MPDQ total score	.59**	1							
3. IAT: Withdrawal and social problems	.94**	.52**	1						
4. IAT: Time management and performance	.86**	.51**	.71**	1					
5. IAT: Reality substitute	.77**	.50**	.69**	.57**	1				
6. MPDQ: Compulsive text messaging	.43**	.82**	.39**	.36**	.37**	1			
7. MPDQ: Compulsive making/receiving calls	.51**	.85**	.44**	.47**	.42**	.48**	1		
8. MPDQ: Distorted thinking	.53**	.80**	.49**	.44**	.48**	.49**	.61**	1	
9. Education level	-.11**	-.20**	-.07*	-.10**	-.10**	-.12**	-.24**	-.12**	1

IAT = Internet Addiction Test, MPDQ = Mobile Phone Dependence Questionnaire.

* Correlation is significant at the 0.05 level (2-tailed).

** Correlation is significant at the 0.01 level (2-tailed).

The pattern of correlations between the subscales of the IAT and the MPDQ provided support for the first five hypotheses. Hypothesis 1: There was a positive correlation between the withdrawal and social problems subscale of the IAT and the compulsive text messaging subscale of the MPDQ, *r* (1072) = .39, *p* < .001. Hypothesis 2: There was significant positive correlation between withdrawal and social problems on the IAT and compulsive making and receiving of calls on the MPDQ, *r* (1072) = .44, *p* < .001. Hypothesis 3: There was a significant positive correlation between time management and performance on the IAT and compulsive text messaging on the MPDQ, *r* (1072) = .36, *p* < .001. Hypothesis 4: There was a significant positive correlation between time management and performance on the IAT and making and receiving calls on the MPDQ, *r* (1072) = .47, *p* < .001. Hypothesis 5: There was a significant positive correlation between reality substitute and distorted thinking, *r* (1072) = .48, *p* < .001.

### Correlations in different age groups

Hypothesis 6 stated that the full-sample correlations between the IAT and MPDQ, and among the subscales of the two scales, would also be evident in the primary school and secondary school subsamples when considered separately. This hypothesis was supported. All subscales showed the expected significant associations, both in the younger students and in the older students (see Tables 5 and 6).

**Table 5:** Correlation matrix of IAT and MPDQ for primary school students (N = 1072).

	1	2	3	4	5	6	7	8
1. IAT total score	1							
2. MPDQ total score	.60**	1						
3. IAT: Withdrawal and social problems	.95**	.54**	1					
4. IAT: Time management and performance	.86**	.56**	.73**	1				
5. IAT: Reality substitute	.75**	.49**	.68**	.57**	1			
6. MPDQ: Compulsive text messaging	.48**	.88**	.42**	.45**	.38**	1		
7. MPDQ: Compulsive making and receiving calls	.56**	.89**	.50**	.54**	.44**	.63**	1	
8. MPDQ: Distorted thinking	.54**	.83**	.49**	.47**	.46**	.60**	.67**	1

IAT = Internet Addiction Test, MPDQ = Mobile Phone Dependence Questionnaire.

** Correlation is significant at the 0.01 level (2-tailed).

**Table 6:** Correlation matrix of IAT and MPDQ for secondary school students (N = 1072).

	1	2	3	4	5	6	7	8
1. IAT total score	1							
2. MPDQ total score	.55**	1						
3. IAT: Withdrawal and social problems	.93**	.49**	1					
4. IAT: Time management and performance	.86**	.45**	.68**	1				
5. IAT: Reality substitute	.77**	.50**	.68**	.55**	1			
6. MPDQ: Compulsive text messaging	.39**	.80**	.35**	.29**	.35**	1		
7. MPDQ: Compulsive making and receiving calls	.44**	.80**	.38**	.40**	.38**	.37**	1	
8. MPDQ: Distorted thinking	.50**	.77**	.46**	.40**	.46**	.42**	.54**	1

IAT = Internet Addiction Test, MPDQ = Mobile Phone Dependence Questionnaire.

** Correlation is significant at the 0.01 level (2-tailed).

### Confirmatory factor analysis (CFA)

CFA was used to test whether there were differences between the IAT factor structure identified in the three subgroups based on grade. First we divided the students into three groups: senior primary, junior secondary, and senior secondary school students. However, as the subsample size for the senior secondary students was only 88, the subsample was likely not representative of this age group. As a result, we categorized the age groups as primary and secondary school students. The results indicated that the difference in CFI between the models for the primary group and the secondary group was 0.90−0.91 = 0.01, indicating multi-group invariance. The goodness-of-fit index (χ2) increased non-significantly (Δχ2 /Δdf < 3, *p* > .05), which indicated that the items used to measure IAT subtypes were statistically equivalent for primary and secondary students in this Hong Kong sample (see Tables 7 and 8).

**Table 7:** Confirmatory factor analysis on primary and secondary school students (N = 1072).

Model	N	χ^2^	df	CFI	TLI	RMSEA
Primary	574	395.901	129	0.905	0.888	0.060
Secondary	504	302.147	129	0.912	0.897	0.052

**Table 8:** Standard deviation level of IAT and MPDQ (N = 1072).

Scale or Subscale	± 1 SD	± 2 SD
1. IAT total score	14.1%	5.7%
2. MPDQ total score	16.2%	3.7%
3. IAT: Withdrawal and social problems	13.9%	4.9%
4. IAT: Time management and performance	14.3%	5.2%
5. IAT: Reality substitute	13.2%	4.5%
6. MPDQ: Compulsive text messaging	16.0%	4.6%
7. MPDQ: Compulsively make/receive calls	16.9%	2.5%
8. MDPQ: Distorted thinking	11.9%	4.2%

SD = standard deviation, MPDQ = Mobile Phone Dependence Questionnaire, IAT = Internet Addiction Test.

### Similarity of distributions

The hypothesized correlations among the IAT and MPDQ total scores and subscales, and the subsequent CFA, provided strong evidence of concurrent validity. Concurrent validity is also reflected in the similarity of the score distributions of the two total scores and of conceptually related subscales from the IAT and the MPDQ. Here, we focused on the percentage of students whose scores were significantly above or below average, indicating either a high or low level of problems associated with Internet addiction or a high or low level of problems associated with mobile phone addiction. For instance, 14% of participants had IAT scores ≥ 1 SD from the mean, and 5.7% had scores ± 2 SD from the mean, values that were similar to those of the MPDQ (16.2% and 3.7%). Similar patterns can be seen in IAT subscales and MPDQ subscales that were hypothesized to be correlated. For example, 13.9% of participants had IAT withdrawal and social problems subscale scores ≥ 1 SD from the mean, and 5% had scores ≥ 2 SD from the mean; on the MPDQ compulsive text messaging scale, the corresponding rates were 16.0% and 4.6%, respectively (see Tables 8–10).

**Table 9:** Standard deviation level of IAT and MPDQ in male participants.

Scale or Subscale	± 1 SD	± 2 SD
1. IAT total score	14.4%	6.1%
2. MPDQ total score	15.0%	3.3%
3. IAT: Withdrawal and social problems	14.2%	4.8%
4. IAT: Time management and performance	14.8%	5.6%
5. IAT: Reality substitute	15.1%	4.7%
6. MPDQ: Compulsive text messaging	15.1%	5.0%
7. MPDQ: Compulsively make/receive calls	19.0%	2.2%
8. MDPQ: Distorted thinking	12.8%	4.6%

SD = standard deviation, MPDQ = Mobile Phone Dependence Questionnaire, IAT = Internet Addiction Test.

**Table 10:** Standard deviation level of IAT and MPDQ in female participants.

Scale or Subscale	± 1 SD	± 2 SD
1. IAT total score	14.8%	5.3%
2. MPDQ total score	14.3%	4.5%
3. IAT: Withdrawal and social problems	13.6%	5.8%
4. IAT: Time management and performance	16.6%	4.5%
5. IAT: Reality substitute	19.3%	5.8%
6. MPDQ: Compulsive text messaging	17.6%	5.8%
7. MPDQ: Compulsively make/receive calls	18.8%	2.0%
8. MDPQ: Distorted thinking	14.1%	4.5%

SD = standard deviation, MPDQ = Mobile Phone Dependence Questionnaire, IAT = Internet Addiction Test.

### Latent class analysis (LCA)

To identify subgroups of persons based on their responses to the IAT, we used the poLCA package to run Latent Class Analysis (LCA) and obtained the results shown in Table 11. LCA is a generally used statistical approach to classify a set of discrete, mutually exclusive latent classes of individuals who are similar in their responses to measured variables [42]. We entered all three IAT subscales into the LCA. The AIC, BIC and χ^2^ are common indices used for model selection, with lower values indicating greater goodness of fit. The BIC is usually more appropriate for basic LCA models due to their relative simplicity. Therefore, BIC and χ^2^ were used as goodness of fit indices in the current study. Table 9 summarizes the results of each model. It can be seen that the 4-class model had the smallest BIC value, but the largest χ^2^ value. The 5-class model had a relatively large BIC but also a relatively small χ^2^. Nevertheless, these two models were more appropriate than the 2- and 3-class models.

**Table 11:** Latent class analysis (N = 1072).

Model	Estimated class population shares	AIC	BIC	χ^2^
2 classes	0.4949 (class 1) 0.5051 (class 2)	23835.31	24039.61	2104100
3 classes	0.2598 (class 1) 0.2638 (class 2) 0.4765 (class 3)	23281.81	23590.75	1985376
4 classes	0.1248 (class 1) 0.1695 (class 2) 0.3435 (class 3) 0.3622 (class 4)	23107.09	23520.66	2487711
5 classes	0.1645 (class 1) 0.1769 (class 2) 0.1231 (class 3) 0.3645 (class 4) 0.1710 (class 5)	23065.39	23583.60	1796446

### Receiver operating characteristic (ROC) analysis

Receiver operating characteristic (ROC) analysis is the standard method for assessing investigative accuracy. The goal of an ROC curve analysis is to determine related cut scores, and as such, it is a beneficial way to interpret sensitivity levels. In this study we used the ROC curve analyses to test the specificity level for IAT and MPDQ in different age groups (primary school, junior secondary school). The ROC analysis suggested that both IAT and MPDQ have a fair sensitivity toward differences between age groups. The results are shown in Table 12.

**Table 12:** Results of ROC curve analysis for IAT and MPDQ.

Test Result Variable(s)	Area	Std. Error	Sig.
IAT total score	.627	.018	.000
MPDQ total score	.719	.017	.000
MPDQ: Compulsive text messaging	.601	.018	.000
MPDQ: Compulsively make/receive calls	.759	.016	.000
MPDQ: Distorted thinking	.658	.018	.000
IAT: Withdrawal and social problems	.599	.018	.000
IAT: Time management and performance	.609	.018	.000
IAT: Reality substitute	.602	.018	.000

ROC = receiver operating characteristic, MPDQ = Mobile Phone Dependence Questionnaire, IAT = Internet Addiction Test.

## Discussion

The overuse of the Internet and smart phones has arisen as a significant problem in many societies. Internet addiction and mobile phone addiction can both be considered forms of technological addiction [23]. Griffiths [43] explained that technological addiction is a behavioral addiction that includes human-machine communication and is non-chemical in nature. The IAT is the most popular assessment tool for Internet addiction [24]. Additionally, the MPDQ has wide application in Asian countries as a measurement tool for mobile phone addiction [16, 33]. This is the first study to document the concurrent reliability of the two measures. The expected moderate correlations suggest that there is some overlap in what is being assessed, but also that each measure assesses a unique form of technological addiction.

Although an increasing number of studies are using the IAT and MPDQ to assess the severity of Internet and mobile phone addiction in adolescents, no studies have established the concurrent validity of these two measures in this population. Concurrent validity was established. The IAT was moderately associated with the MPDQ, an already validated measure of mobile phone addiction, when the two measures were administered at approximately the same time. This evidence can be used to support the use of these assessments as a way to recognize children and adolescents’ addictive behavior regarding Internet and mobile use.

### Developmentally appropriate measures

Apart from the concurrent validity of the IAT and MPDQ, the current study also found that the IAT and MPDQ are developmentally appropriate for primary students and secondary students in Hong Kong. Previous studies in Hong Kong usually applied only the IAT [10,11,12] or MPDQ [31] to evaluate technological addiction in adolescents. These local studies seldom tested whether the MPDQ and IAT seldom tested whether the MPDQ and IAT had similar psychometric properties in different age groups. Our results showed that there was a significant positive correlation between the three subscales of the IAT and the three subscales of the MPDQ in both primary school and secondary school students. Furthermore, the LCA outcomes provided support for the IAT classification system. Finally, the CFA results showed that the items used to measure IAT subtypes were statistically equivalent for primary and secondary students in Hong Kong.

### Addiction is negatively associated with education level

Some studies in Hong Kong have found that education level is negatively associated with Internet addiction, consistent with the assumption that there is a decrease in Internet addiction as adolescents age [9,10]. In the current study, we found a similar outcome, with education level being negatively correlated with IAT and MPDQ scores. Shek and Yu [10] explained that this situation might be due to cognitive maturation and engagement in other activities, which reduce adolescents’ attention to the Internet. Meanwhile, a highly competitive academic environment begins at the senior level in Hong Kong because of students’ need to prepare for the university entrance exam. Therefore, addiction in students might gradually decrease over time. Nevertheless, these observations suggest that there is a need for further research in order to step up prevention programs for Internet and mobile phone addiction in Hong Kong.

### Development of screening tool

The Hong Kong Federation of Education Workers [31] stated that at least 20% of secondary school students were seriously addicted to the Internet. Shek and Yu [10] showed that almost 26.4% of students met the criteria for Internet addiction in Hong Kong. Meanwhile, Leung [33] indicated that 16.9% of the students fell into the category of high-level mobile dependency. In the current study, we found that ± 1 SD for IAT scores included 14.1% of participants, and for MPDQ the corresponding value was 16.2%. These rates are near to the prevalence rate of Internet addiction and mobile phone addiction in Hong Kong. As a result, both the IAT and MPDQ can be considered screening tools for technological addiction in Hong Kong.

### Further development

The Internet and smart phone technology are rapidly developing in the 21^st^ century, and traditional research methods may not be suitable to understand Internet addiction and mobile phone addiction. One new research method involves collecting data from mobile apps. This “big data” approach will help researchers to understand the development of norms at different ages, such as primary students from age 9 to 12, junior secondary students from 12 to 15, senior secondary students from 15 to 18, and university students and working adults over 18 years old. The classification of information from different age groups can help us to more easily track the variety of differences between age groups and the developmental norms regarding Internet addiction and mobile phone addiction. This will help teachers and parents to identify potentially addicted children and adolescents. On the other hand, big data can store different countries’ statistics and can further help researchers to develop multicultural norms through assessment apps on mobile phones and other technology. Finally, screening apps can also help researchers understand adolescents’ online preference patterns, such as differences between genders in online behavior. Based on this information, researchers and frontline counselors can set up more appropriate prevention and treatment programs for Internet and mobile phone addicted users.

### Limitations

Several limitations may have affected the outcomes of this study. First, all measures were self-reports, which are subject to bias due to social desirability [44]. Future studies should also include information reported by parents and teachers, and information about the specific apps and platforms that youth are using. Secondly, uncontrolled clustering effects happen in this study. Even though we randomly invited the schools join in this research, the student’s arrangement was controlled by the school teachers due to convenient the class management and participants recruitment. Future studies should directly invite the students to fill in the questionnaires in the clinical center or use the apps to submit the answers. Thirdly, the results lack clinical cases for comparison. None of the students in the current study had been diagnosed with Internet or mobile phone addiction. Future studies should also recruit a clinical sample. Additionally, the results of this study are based on an examination of urban Hong Kong primary and secondary school students 9–18 years old. The results may not be valid for other countries, rural students, or children and adolescents not attending school. Finally, a longitudinal design is suggested to explore the long-term influences of Internet and mobile phone addiction on the social and emotional development of adolescents.

## Conclusions

This is the first study to document the concurrent validity of the IAT and the MPDQ, documenting similarities and differences between these measures of Internet addiction and mobile phone addiction, respectively. Furthermore, the IAT and MPDQ were shown to be developmentally appropriate tools for students aged 9 to 18. This study has provided an empirical foundation for documenting the utility of these measures.
